# Graft Survival Rate of Renal Transplantation: A Single Center Experience, (1999-2009)

**Published:** 2011-06-01

**Authors:** A Almasi-Hashiani, A R Rajaeefard, J Hassanzade, H Salahi, S Nikeghbalian, P Janghorban, S A Malek-Hosseini

**Affiliations:** 1 Department of public Health, School of health, Arak University of Medical Sciences, Arak, Iran; 2 Department of Epidemiology, School of Health and Nutrition, Shiraz University of Medical Sciences, Shiraz, Iran; 3 Shiraz Organ Transplantation Center, Nemazee Hospital, Shiraz University of Medical Sciences, Shiraz, Iran

**Keywords:** Renal transplantation, Graft survival, Cox regression model, Iran

## Abstract

**Background:**

Renal transplantation is the best option for treatment of the end-stage renal diseases and has more advantages than dialysis. The objective of this study is to determine the ten-year graft survival rate of renal transplantation and its associated factors in patients who have been transplanted from March 1999 to March 2009 in Nemazee Hospital Transplantation Center.

**Methods:**

This is a historical cohort study of 1356 renal transplantation carried out during 1999 to 2009. Kaplan-Meier method was used to determine the survival rate, log rank test to compare survival curves, and Cox regression model to determine hazard ratios and for modeling of variables affecting survival.

**Results:**

The 1, 3, 5, 7 and 10 years graft survival rates were 96.6, 93.7, 88.9, 87.1 and 85.5 percent, respectively.Cox regression model revealed that the donor source and creatinine level at discharge were effective factors in graft survival rate in renal transplantation.

**Conclusion:**

Our study showed that 10 year graft survival rate for renal transplantation in Nemazee Hospital Transplantation Center was 85.5% and graft survival rate was significantly related to recipients and donor’s age,donor source and creatinine level at discharge. Our experience in renal transplantation survival rate indicates asuccess rate comparable to those noted in other reports.

## Introduction

In the western world, using renal replacement therapy (dialysis and transplantation) for patients with endstage renal disease (ESRD) is going to be increased while the same epidemiological trend is also being seen in other countries and so is Iran.[1][2] In year 2006,there were 25000 patients with ESRD who were under renal-replacement therapy in Iran and considering an annual growing rate of 12%, number can reach to 40000 patients by the year 2011. The prevalence rate of ESRD has been reported to be 357 cases per one million populations per year.1 These patients have been treated by dialysis and kidney transplantation.[3][4][5][6] Kidney transplantation was the treatment of choice for ESRD[7][8] that restored the patients' quality of life and reduced the morbidity and mortality rates.[9][10] Allograft rejection is the most important complication that reduces the graft survival after transplantation.Many variables contribute in survival or rejection of graft, such as donor source, age and gender, creatinine level, blood group, Rh type, waiting time, duration of hospitalization, vascular complications and acute rejection. The aim of this study was to determine the organ survival rate after kidney transplantation during a period of 10 years (1999-2009) in Shiraz Transplant Center, Nemazee Hospital, Shiraz, Southern Iran.

## Materials and Methods

This historical cohort study was intended to consider the graft survival rate in all patients (1356 cases) who received kidney transplantation in Shiraz Transplant Center, Nemazee Hospital, Shiraz, Southern Iran, during a period of 10 years from March 1999 to March 2009. The precise time of transplantation was considered to be the "initial event" and when renal allograft was diagnosed to be completely and irreversibly non-functioning due to any cause including rejection and the patient requirements regular dialysis again, was defined as "end-point event".

We have not been performing preoperative donor conventional or computed tomography (CT) angiography during the last 6 years of this period, and duplex ultrasonography and intravenous pyelography are the only imaging studies of the renal system of the donors that we used. CT angiography was used only when the duplex ultrasonography suggested any abnormal findings in renal vasculature. This selective use of contrast imaging studies prevents the use of excessive doses of intravenous contrast agents which may be toxic for the donor kidneys and warrants the safety of the donor surgery and picks up an occasional donor with renovascular pathology. We prefer left kidney because of longer renal vein and better accessibility for nephrectomy and use the right kidney only in special situations.

The studied variables compromised donor's and recipient's age, gender (male/female and same or compatible), blood group (ABO and same or compatible), and recipient's immunosuppressive drug regimen, recipient's job, underlying cause of ESRD, donor source, time of first urination, vascular complication, endarterectomy, allograft warm and cold ischemic times, creatinine level at discharge and the duration of dialysis therapy before and hospital stay after transplantation. We used intravenous methylprednisolone for induction of immunosuppressive regimen for all patients. Four different regimens had been prescribed to recipients for maintenance immunosuppressive regimen including i) Oral Prednisolone, Azathioprine (Imuran®) and Cyclosporine (Neoral®), ii) Oral prednisolone, mycophenolate mofetil (Cell-cept®) and cyclosporine (Neoral®), iii) Oral prednisolone, azathioprine which was changed to mycophenolate mofetil (Cellcept®) after different time intervals and cyclosporine (Neoral®) and iv) Oral prednisolone, mycophenolate mofetil (Cell-cept®) and tacrolimus (Prograf®).

All needed data were collected through reviewing of patients' hospital records. The organ survival and every patient's need to regular dialysis were assessed and determined by nephrologists and recorded in follow-up clinics and related institutions such as "Management Center for Transplantation and Special Diseases" and "Renal Patients Support Society". Allograft survival rate was calculated by Kaplan-Meier method and Log-rank test was used to compare survival curves and Cox Regression Models to define the hazard ratio and for modeling of factors implicating in survival rate. The SPSS® software (version16, Chicago, IL, USA) and STATA (version 9 for assessment proportionality of hazard ratio assumption) were used for statistical analysis of data. The P-value of less than 0.05 was considered to be statistically significant.

## Results

One thousands and three hundred and fifty six cases of kidney transplantation were performed in Shiraz Transplant Center, Nemazee Hospital, during a period of 10 years (1999-2009). Out of these, 403 (29.7%) kidneys were from living related donors, 441 (32.5%) kidneys from living unrelated donors and 512 (37.8%) kidneys from deceased donors. We could follow 1288 (94.9%) out of 1356 patients who had received kidney and found that 107 (7.9%) of them had been eventuated in irreversibly non-functioning allografts that necessitated regular dialysis again. The mean age of kidney donors and recipients were 34.99±13.94 and 31±11.21 years, respectively. The mean duration of hospital stay and duration of dialysis before operation were 12.29±5.63 days and 15.93±14.52 months, respectively, and mean followup period was 47.23±33.33 months.

The age of donors and recipients in 94.5 and 84.2% of cases were less than 50 years old, respectively. 64.4 percent of recipients were male and 35.6% were female. The males and females comprised 65.9 and 34.1% of donors respectively, and 41.7% of transplantations were performed from male to male. Blood type of “O” has been the most frequent blood type among recipients and donors with 43.3 and 50.4% of cases respectively, and blood group and Rh of donor and recipient in 85% of cases were identical. Considering creatinine level status at discharge, in 85.5% of recipients was 2 mg/dl or less. Warm and cold ischemia time in 100 and 57.6% of cases were less than 2 hours, respectively. Endarterectomy was not performed in 92.1% of cases and in 92.3% of cases; the first time to urination was immediate. In 61.7% of cases, the duration of hospitalization after operation was from 7 to 14 days. The underlying cause of ESRD was mostly unknown (comprising 53.9% of cases). However, the most common diagnosed renal disease leading to ESRD was glomerulonephritis (in 27.7% of cases). The first regimen was the most frequently used immunosuppressive therapy which had been administered in nearly 49.3% of cases.

**Figure 1:**
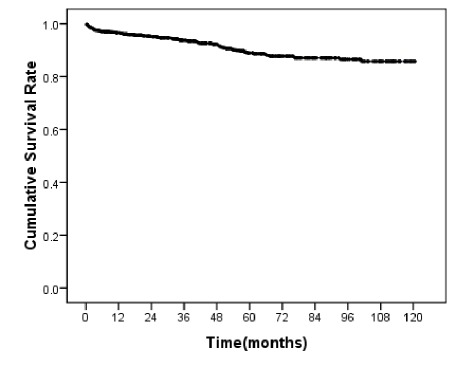
Allograft survival rate in renal transplant recipients.

As shown in Figure 1, the calculated survival rate of 1, 3, 5, 7, 10 year for renal transplantation using Kaplan-Meier method was 96.6, 93.7, 88.9, 87.1 and 85.5%, respectively. For investigating the existence of any significant difference among the different classes of these variables from the point of survival rate, Log-rank method calculated p-values, have been calculated and age of donor (p=0.005), donor source (p=0.001), HCV infection (p=0.029), creatinine level at discharge (p=0.001), and duration of hospitalization after operation (p=0.017) showed significant relationship with renal transplantation graft survival rate. In the other variables, there was no significant difference among their graft survival curves.

**Figure 2:**
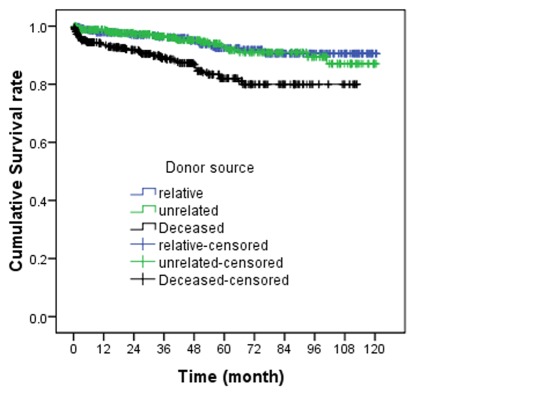
Allograft survival rate in renal transplant recipients based on donor source.

Using Cox proportional hazard model that was shown in Table 1, we found that donor source and creatinine level at discharge (as shown in Figure 2 and Figur 3) had a significant relationship with graft survival rate. Hazard ratio in deceased-donor transplantation compared to living-related donor has been 2.56 (CI=1.36–4.78, p=0.003). Also, in the cases which discharge time, the creatinine level was more than 2 mg/dl in comparison to cases with creatinine level of 2 mg/dl or less and a hazard ratio of 3.97 (CI=2.3–6.8, p=0.001).

**Table 1:** Multivariate analysis by Cox Regression Model

**Variables**	**Hazard ratio**	**P value**	**95 % CI for hazard ratio**	
			**Lower limit**	**Upper limit**
Donor source				
Related	1	-	-	-
Unrelated	1.04	0.8	0.51	2.12
Deceased	2.56	0.003	1.36	4.78
Creatinine at discharge 2≥mg/dl	1	-	-	-
>2 mg/dl	3.97	0.001	2.3	6.8

**Figure 3:**
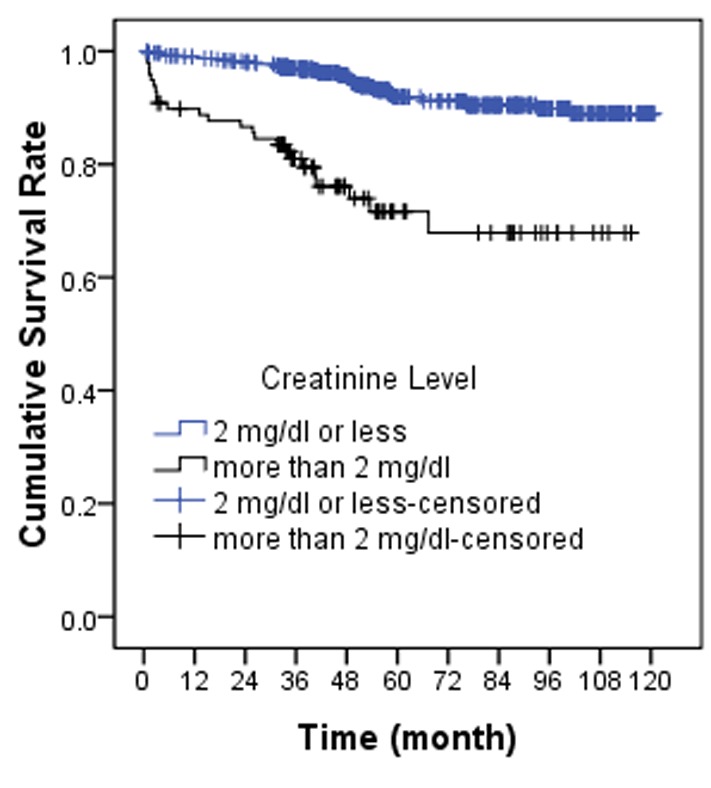
Allograft survival rate in renal transplant recipients based on creatinine level at discharge.

## Discussion

In this study, the survival rate of 1, 3, 5, 7, and 10- years have been 96.6, 93.7, 88.9, 87.1, and 85.8% respectively. According to the report of Iranian Network for Organ Procurement, one- year survival rate in Iran taps around 95%, a result which is similar to our study.

Using Kaplan-Meier method, the age of donor has been known as one of the contributor factor on survival rate. A lot of studies have been conducted upon above mentioned issue which some of them are studies of Briganti[11] and Orsenigo et al.,[12] which showed that the age of donor and recipient are of contributing variables. These results have been confirmed by plenty of other studies,[13][14][15][16][17] but using Cox proportional hazard model we found that the age of donors and recipients were not significant with graft survival rate of renal transplantation.

Many studies have shown that there is no significant relationship between the gender of graft donor and recipient with survival rate of transplantation11,13,17 and our study is another evidence for this correlation. One of the effective variables on survival rate which also has been studied,[11][13] is the donor source (living- related donor, living- unrelated donor and deceased-donor). In this study, this factor was also determined and revealed that the living- donor survival rate is significantly higher in comparison with deceased donor. Albeit that is true, using deceased organs is one of the major strategies for shortening the waiting time for transplantation, following that, the time of pre- operation dialysis which can be one of the important factors on survival rate decreases.

Briganti et al.,[11] Mohamed et al.,[16] and Bruce Kaplan et al.,[14] reported that the increase of cold ischemia time leads to the decrease of transplantation survival rate significantly. On the other hand in the study that Courtney et al.[13] conducted, there was no significant relationship between survival rate and cold ischemia time which is similar to our study findings. One of the reasons for differences in observed results might be the incomplete registration of cold ischemia time in our study, since in this study, only in 55.3% of cases, cold ischemia time was etermined.

In many studies, the graft and patient’s survival rate in HCV positive and negative groups has been reported equal.[18][19][20][21] On the other hand, in some studies the graft survival rate for HCV negative cases in comparison to HCV positive cases have been significantly higher,[16][22] and this study also confirms that graft survival rate in different HCV donors group was not significant.

In this study, the graft survival rate between different occupational groups was not significant. Also, having or not having the history of endarterectomy, iliac type of vein and artery and the number of used veins and arteries (simple, double and triple) have not shown significant relationship with survival rate. A lot of studies, including Courtney et al.'s study,[13] have shown that the cause of ESRD is one of those factors that have this potential to influence the graft survival rate of kidney transplantation. On the other hand some reports have represented that there is no significant relationship between survival rate and the cause of ESRD.[11][23][24] In this study, there was no significant relationship between survival rate and primary renal disease too, albeit this should be mentioned that in over the 50% of the cases (53.9%) the reason of ESRD was not clear.

Cox regression model demonstrated that duration of dialysis before operation had no significant relationship with the survival rate and this finding is similar to other results.[25] On the other hand, in some reports, there was a significant correlation between graft survival rate of transplantation and duration of dialysis before operation.[11][26] The right or left being of donor kidney was from those variables which had not significant relationship with survival rate. In most cases, the left kidney because of having a longer vein was selected for transplantation and in this study composed 98% of the cases among whom, the left or right side of kidney was known.

The duration of post-operation hospitalization is another independent variable which had influence on the survival rate while in the final model had no significant relationship with survival rate. In recent years, there has been a descending trend in the period of patient hospitalization and in the recent decade, the period of hospitalization in renal transplantation has decreased from 12.7-19 days to 5-7.5.[27][28] Lin’s study showed that less than 4 days hospitalization would be one of the possible effective factors of the reduction of survival rate and patient survival in the long run.[28] In recent years, the progress in immunosuppressive regimen has led to improvement of transplantation survival rate and patient’s survival rate, but in this study, no significant relationship between immunosuppressive drug regimen and graft survival rate was noticed and also in other studies, there was no association between immunosuppressive regimen and the survival rate.[26][27][29]

Creatinine level at discharge was another variable which showed a significant relationship with the survival rate of kidney transplantation on Cox model which is similar to Rayhill's et al.'s study,[30] Based on Rayhill's et al.'s study, per one singular unit increased the creatinine level and the transplantation risk ratio increased 1.8 times.

Our study showed that 10 year graft survival rate for renal transplantation in Nemazee Hospital Transplantation Center was 85.5% and graft survival rate was significantly related to recipients and donor’s age, donor source and creatinine level at discharge. Our experience in renal transplantation survival rate indicates a success rate comparable to those noted in other reports.
